# Supplementary material for: Precautions during Direct Oral Anticoagulant Introduction in Gynecologic Malignancies: A Single-Center Retrospective Cohort Study
Source: Cancers (Basel). 2023 Feb 10;15(4):1132. doi: 10.3390/cancers15041132 (PMC9954552; doi:10.3390/cancers15041132)
Supplement: Supplementary file 1 [file cancers-15-01132-s001.zip › Table S1.pdf]

Table S1. Difference in cause-specific RMTL in each risk factor for a primary outcome*

Risk factors for a primary outcome	RMTL (95% CI), days	Difference in RMTL for primary outcome (95% CI), days	*P*-value
**Location of blood clots when VTE was first diagnosed**			
PE or proximal DVT without PE	170 (74－266)	170 (74－266)	0.001
Isolated distal DVT	0.00 (NA)**	Reference	NA
**D-dimer level when VTE was first diagnosed**			
Tertile 1 or 2 (<7.6 μg/dL)	27 (-8－62)	Reference	NA
Tertile 3 (≥7.6 μg/dL)	238 (94－382)	211 (63－359)	0.005

*RMTLs for clear cell carcinoma of the ovary could not be estimated.

**No patients with Isolated distal DVT for a primary outcome

Abbreviations: CI, confidence interval; DOAC, direct oral anticoagulant; DVT, deep vein thrombosis; NA, not applicable; PE, pulmonary embolism; RMTL, restricted mean time lost; VTE, venous thromboembolism.
