# Supplementary figures and images for: Precautions during Direct Oral Anticoagulant Introduction in Gynecologic Malignancies: A Single-Center Retrospective Cohort Study
Source: Cancers (Basel). 2023 Feb 10;15(4):1132. doi: 10.3390/cancers15041132 (PMC9954552; doi:10.3390/cancers15041132)

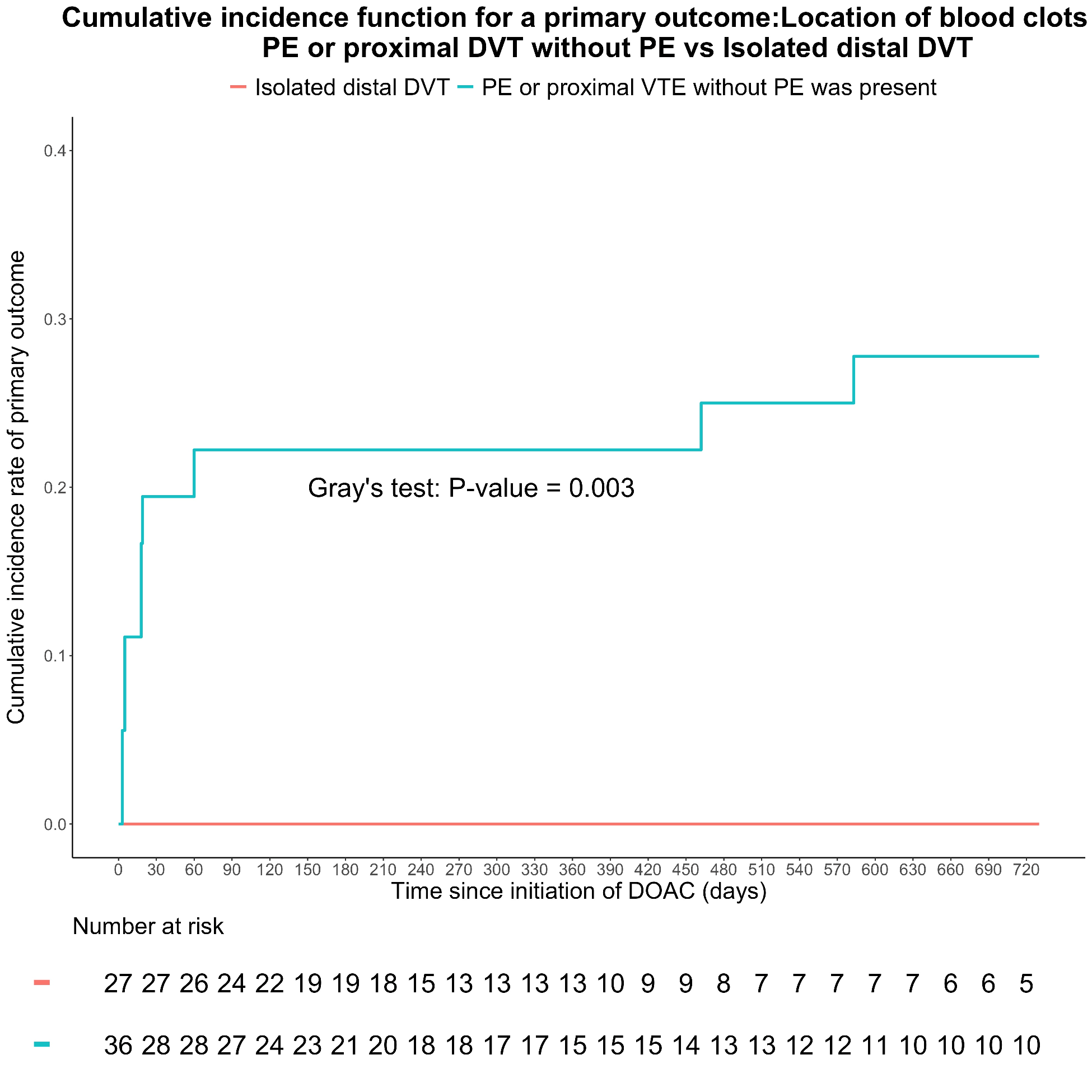

Supplement: Supplementary file 1 [file cancers-15-01132-s001.zip › Figure S1.tif]

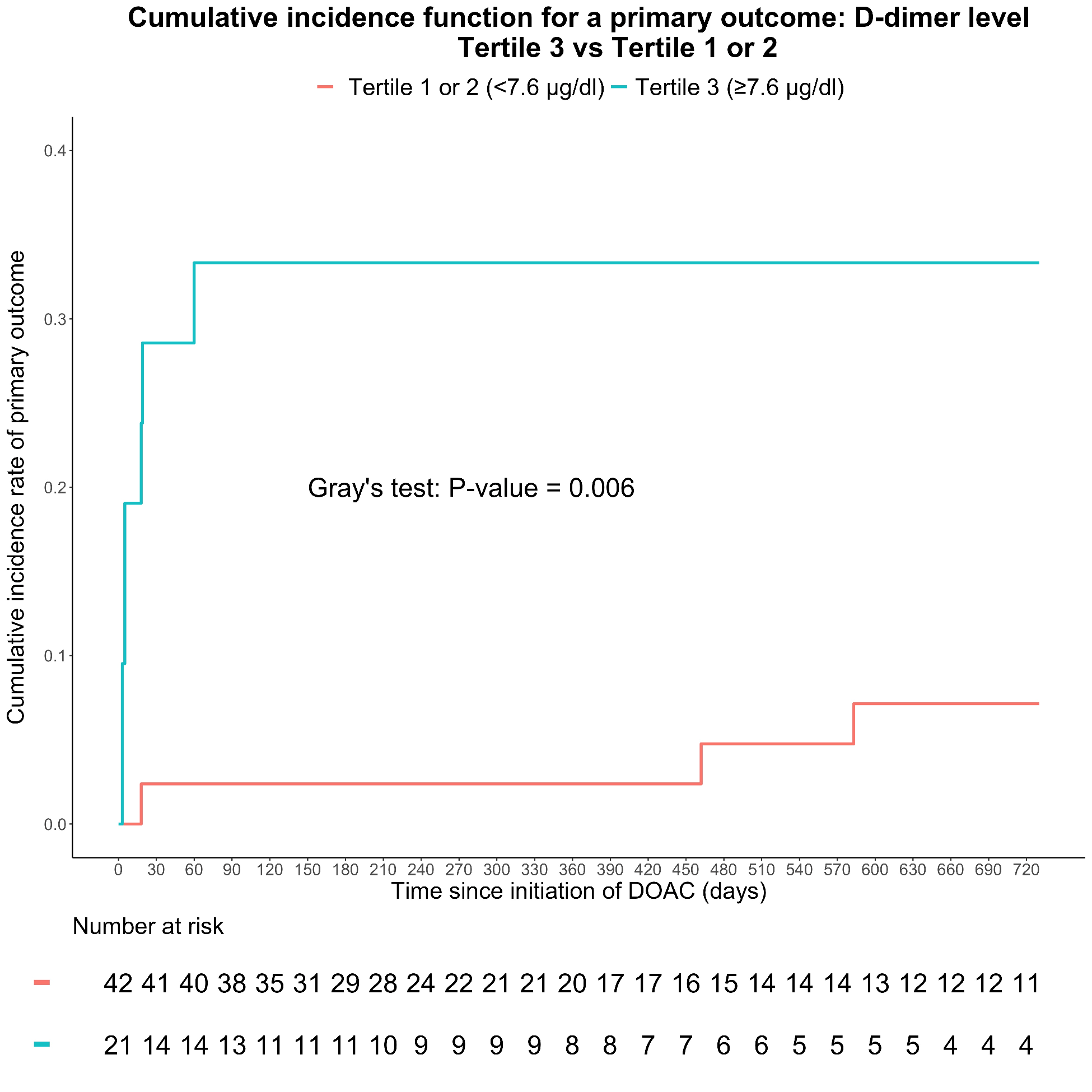

Supplement: Supplementary file 1 [file cancers-15-01132-s001.zip › Figure S2.tif]

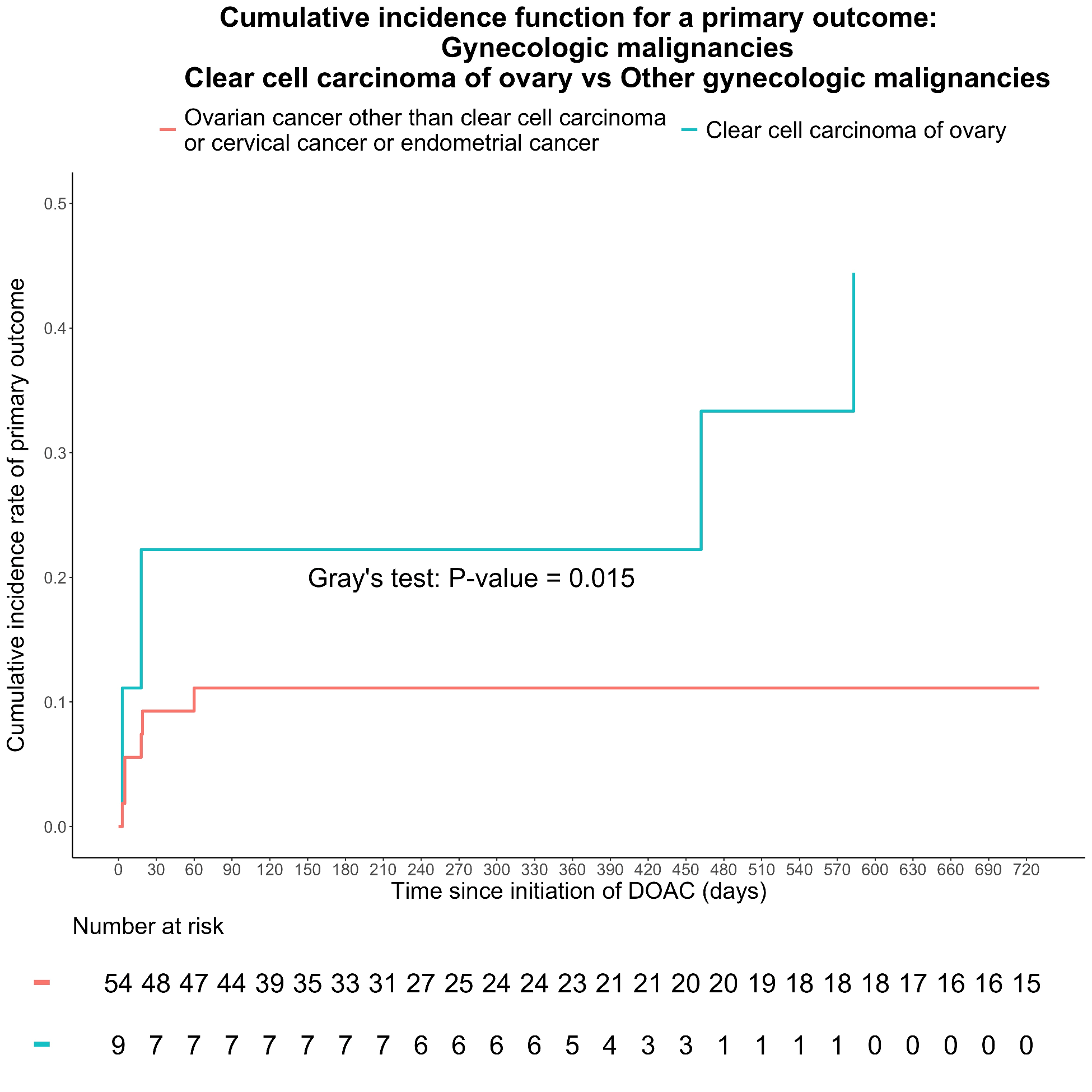

Supplement: Supplementary file 1 [file cancers-15-01132-s001.zip › Figure S3.tif]
